# Comparative Tuberculosis (TB) Prevention Effectiveness in Children of Bacillus Calmette-Guérin (BCG) Vaccines from Different Sources, Kazakhstan

**DOI:** 10.1371/journal.pone.0032567

**Published:** 2012-03-09

**Authors:** Michael Favorov, Mohammad Ali, Aigul Tursunbayeva, Indira Aitmagambetova, Paul Kilgore, Shakhimurat Ismailov, Terence Chorba

**Affiliations:** 1 International Vaccine Institute, Seoul, Korea; 2 Centers for Disease Control and Prevention, Atlanta, Georgia, United States of America; 3 Central Asia Office, Centers for Disease Control and Prevention, Almaty, Kazakhstan; 4 Kazakhstan National Tuberculosis Center, Almaty, Kazakhstan; University of Otago, New Zealand

## Abstract

**Background:**

Except during a 1-year period when BCG vaccine was not routinely administered, annual coverage of infants with Bacillus Calmette-Guérin (BCG) in Kazakhstan since 2002 has exceeded 95%. BCG preparations from different sources (Japan, Serbia, and Russia) or none were used exclusively in comparable 7-month time-frames, September through March, in 4 successive years beginning in 2002. Our objective was to assess relative effectiveness of BCG immunization.

**Methods/Findings:**

We compared outcomes of birth cohorts from the 4 time-frames retrospectively. Three cohorts received vaccine from one of three manufacturers exclusively, and one cohort was not vaccinated. Cohorts were followed for 3 years for notifications of clinical TB and of culture-confirmed TB, and for 21 months for TB meningitis notifications. Prevention effectiveness based on relative risk of TB incidence was calculated for each vaccinated cohort compared to the non-vaccinated cohort.

Although there were differences in prevention effectiveness observed among the three BCG vaccines, all were protective. The Japanese vaccine (currently used in Kazakhstan), the Serbian vaccine, and the Russian vaccine respectively were 69%, 43%, and 22% effective with respect to clinical TB notifications, and 92%, 82%, and 51% effective with respect to culture confirmed TB. All three vaccines were >70% effective with respect to TB meningitis.

**Limitations:**

Potential limitations included considerations that 1) the methodology used was retrospective, 2) multiple risk factors could have varied between cohorts and affected prevention effectiveness measures, 3) most cases were clinically diagnosed, and TB culture-positive case numbers and TB meningitis case numbers were sparse, and 4) small variations in reported population TB burden could have affected relative risk of exposure for cohorts.

**Conclusions/Significance:**

All three BCG vaccines evaluated were protective against TB, and prevention effectiveness varied by manufacturer. When setting national immunization policy, consideration should be given to prevention effectiveness of BCG preparations.

## Introduction

### Background

Since 1921, Bacillus Calmette-Guérin (BCG) vaccine has been given to infants to reduce the risk of tuberculosis (TB) disease, and disseminated TB [1]. Generally, prospective randomized trials of BCG have been used to evaluate efficacy of BCG, and retrospective case-control studies of BCG have been used to compare effectiveness of different BCG vaccine strains. Although measures of BCG prevention effectiveness/efficacy have not been consistent [2], when BCG immunization of newborns was stopped in Sweden, a circumstance that provided a non-vaccinated comparison group, a six-fold increase in TB notifications was observed in infants [3]. In a prospective randomized control trial in Britain in which over 50,000 older children were allocated to no vaccination or one of two vaccine groups, comparable prevention efficacy of 81% to 84% was found among those vaccinated with BCG (*Mycobacterium bovis*) or with vole bacillus (*Mycobacterium microti*), respectively, when data were compared over a 20-year period [4]. However, in another randomized control trial in India in which over 100,000 uninfected subjects with a normal chest radiograph were allocated to placebo or one of four vaccine groups, no difference was observed in TB incidence among cohorts immunized with a placebo, a low-dose or high-dose Danish BCG strain, or a low-dose or high-dose French BCG strain [5]. In an extensive review in 1983 of eight prospective BCG trials (in the majority of which positive prevention efficacy with BCG was found), the two trials that rated highly in all examined aspects of methodological and statistical quality reported prevention efficacy for TB of 76% or more [6]. In a later meta-analysis of 14 prospective trials, prevention efficacy for TB for BCG recipients was 51% (95% confidence interval [CI], 30% to 66%) [1]. In the same meta-analysis, seven trials reporting TB deaths showed prevention efficacy of BCG vaccine of 71% (95% CI, 47% to 84%), and five trials reporting on meningitis showed prevention efficacy of BCG vaccine of 64% (95% CI, 30% to 82%) [1]. Differences in relative prevention effectiveness between BCG preparations have been described in two case-control studies with different vaccines, but these studies have lacked non-vaccinated comparison groups for estimating absolute effectiveness [7], [8].

**Figure 1:**
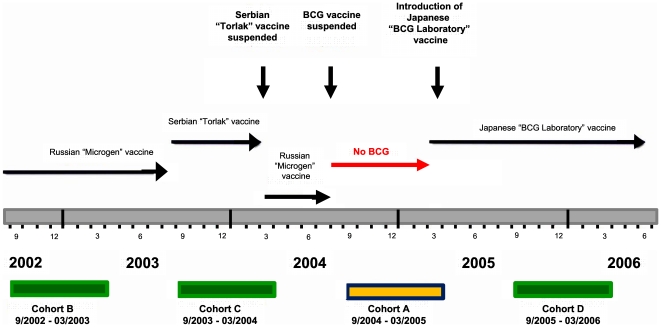
Cohorts of vaccinated and non-vaccinated children in Kazakhstan, 2002–2006.

From the mid-1960s until 2003, BCG vaccine produced in Russia was administered routinely at birth to all infants in Kazakhstan. Since 2001, in years when there has been a BCG immunization program in place for the full 12 months, reported BCG coverage of infants in Kazakhstan has ranged from 95% in 2001, to 99% in 2002, 2003, 2006, and 2007 [9]; since 1997, the published World Health Organization estimates of BCG coverage have been consistent with the official reported BCG coverage rates for Kazakhstan [9]. Although administration mode data are not gathered routinely, the standard practice in Kazakhstan is intradermal delivery of this vaccine. Beginning in March 2003, the Government of Kazakhstan gradually changed its BCG vaccine procurement source from its Russian source,–Microgen, the Federal State Scientific-Industrial Company for Immunobiological Medicines, an enterprise of the Ministry of Health (MOH) of the Russian Federation, to a Serbian source,–Torlak Institute of Immunology and Virology, a global producer of vaccines and biopharmaceuticals in Belgrade. The change in source was associated with 1,282 reports of adverse post-vaccination events in newborns in 2003; of these, 1,260 (98%) were reports of lymphadenitis. A subsequent investigation yielded reported cases of lymphadenitis in 1.5% (26/1,747) of children vaccinated with the Serbian BCG compared to 0.02% (1/4,217) of children vaccinated with the Russian BCG (RR = 62.6 (95% CI: 8.5–462.1); p<0.001) (Kazakhstan MOH, unpublished data). As a result, the Kazakhstan MOH issued a decree in February 2004 suspending use of the Serbian BCG vaccine. The Russian BCG continued to be administered to fewer than 5% of newborns, but after reports of adverse events in 54 children given the Russian BCG between May and July 2004, the MOH completely suspended the use of BCG vaccine on July 30, 2004. From August 2004 to March 2005, the BCG vaccination program in Kazakhstan was mostly disbanded (Figure 1). As a consequence, the reported BCG coverage of infants in Kazakhstan decreased to 65% in 204 and 69% in 2005 [9]. However, in mid-March 2005, infant vaccination with BCG resumed, with vaccine produced by Japan BCG Laboratory (Tokyo), and use of this vaccine has continued into 2011. For the cohort that was not vaccinated with BCG in 2004–2005, there was no national “catch-up” vaccination campaign. Nevertheless, some oblasts provided immunization for free for non-vaccinated children in response to parental request; however, these measures were not systematic.

### Objective

Because assessing differences in relative effectiveness of different BCG vaccines would be of value in allocating resources in resource-constrained countries where TB is highly endemic, the objective of this study was to assess whether there were differences in the relative prevention effectiveness of BCG vaccines produced by three different manufacturers and administered from 2002 through 2006 in the context an intradermal BCG vaccination program in which there were no major identifiable changes other than the addition or subtraction of specific BCG vaccines.

## Methods

### Overall

We used vaccination, TB notifications, and laboratory data for four different cohorts of children. Three cohorts had received one of the three BCG preparations intradermally, and one cohort had received no BCG. Each cohort was followed over comparable time-frames in successive years using three different TB reporting models. The first model included clinically-defined, radiologically-confirmed cases, the second was restricted to laboratory-confirmed cases, and the third was restricted to reported cases of TB meningitis. Because it was possible for a given case to be in more than one model, there was overlap between the models.

### Populations Studied and Ethics Approval

National TB data were obtained from aggregated surveillance statistics published annually by the Kazakhstan National Tuberculosis Center [10], and as such, this activity was determined to be program evaluation and not human subjects research. General population estimates, and estimates of the number of children in successive cohorts were obtained from publications of the Republic of Kazakhstan National Statistics Agency [11]. All personal information was removed from national TB surveillance data before analysis. In addition, human subjects review and ethical approval for these analyses were obtained from the U.S. Centers for Disease Control and Prevention (CDC), which determined that this project was program evaluation and not human subjects research.

### Data Sources and Overall TB Rates

The Kazakhstan MOH has included surveillance strengthening as an important component of its directly observed therapy, short-course (DOTS) program implementation. As a joint public health program development effort, national case-based, disease-specific electronic surveillance was first implemented in 1998 by the MOH, with the support of CDC and the U.S. Agency for International Development (USAID), to collect and analyze data necessary to monitor and evaluate key TB program activities. Development and adaption of Kazakhstan's TB Electronic Surveillance and Case Management (ESCM) system have included development of electronic software in compliance with WHO requirements for national case-based surveillance. The ESCM system was evaluated jointly by the MOH and CDC in 2003 and 2006, and comparisons of completeness of reporting of the electronic and paper-based surveillance systems favored the electronic system, supporting discontinuation of traditional paper-based surveillance and continuation of the ESCM.

In this study, published aggregated/unlinked data were used from the ESCM system from 2002–2009, and included notifications of 300,098 TB cases. The following variables were used in the analysis: date of birth, date of case notification, radiological confirmed TB disease, TB meningitis, culture of sputum or gastric aspirate, and laboratory confirmation of TB (culture positivity). Data collection was performed by the Government of Kazakhstan. CDC and the International Vaccine Institute participated collaboratively with the Government of Kazakhstan in study design, analysis, interpretation of data, and decision to submit the work for publication.

### Study Design

This study was a retrospective comparison of outcomes of four different birth cohorts. For the period 2002–2006, outcomes of three cohorts of BCG-vaccinated children (September 2002–March 2003, September 2003–March 2004, September 2005–March 2006) and one cohort of non-vaccinated children (September 2004–March 2005) were analyzed. Births and inclusion in each new cohort began on September 1 of a given year and ended on March 31 of the subsequent year (Figure 1). The cohorts and follow-up periods are described in Table 1. We estimated the number of births for the 7-month cohorts as: [(the number of live births in each year of the study÷12)×4]+[(number of live births in the following year÷12)×3]; thus, a 4-month estimate of the number of live births from September through December in any given year was added to a 3-month estimate of the number of live births from January through March in the subsequent year. The 7-month inclusion period for each cohort was succeeded immediately by a 29-month follow-up period during which time the cohort was followed, yielding a total potential cohort inclusion and follow-up time of 3 years. The respective cohorts were followed for the full 3 years for TB incidence, both for notification of clinical or culture-positive cases, and for 21 months for notifications of TB meningitis. Hence, the numbers of clinical cases and the numbers of culture-positive cases for the different cohorts during the follow-up periods included all cases diagnosed in a cohort between the first day of the cohort inclusion period and a final day 3 years later, and for TB meningitis, the cases diagnosed in a cohort between the first day of the cohort inclusion period and a day 21 months later.

**Table 1:** Birth cohorts, BCG vaccine used, inclusive months of cohort entry, and length of follow-up, Kazakhstan, 2002–2008.

Cohort (vaccine used) and number of newborns in cohort	Inclusive months of cohort entry (births)	Inclusive months of follow-up period
A (no BCG vaccine) n = 160,970	Sep 2004–Mar 2005	Sep 2004–Aug 2007
B (“Microgen”, Russia) n = 138,059	Sep 2002–Mar 2003	Sep 2002–Aug 2005
C (“Torlak”, Serbia) n = 150,938	Sep 2003–Mar 2004	Sep 2003–Aug 2006
D (“BCG laboratory”, Japan) n = 168,664	Sep 2005–Mar 2006	Sep 2005–Aug 2008

Laboratory-confirmed data were based on TB culture results routinely reported to the ESCM system by TB hospital laboratories. Because these were strictly surveillance data, no attempt was made to standardize the reports among oblasts for scientific analyses, for variation because of equipment limitation, or for variations in training levels of laboratory personnel. Because of diagnostic challenges and difficulties inherent in sputum collection in the pediatric population, we also examined TB notification data for older children and adults (persons aged 15–44 years and 45+) for the years 2002–2007 to determine if reported incidence rates changed in this population, in the event that factors other than changes in BCG vaccine type could account for any changes in incidence observed in the four birth cohorts.

Because there was no national “catch-up” immunization campaign for the non-vaccinated cohort, data regarding the numbers of children in this cohort who were subsequently vaccinated with BCG were not systematically gathered and were not available for analysis.

### Analysis

To assess BCG effectiveness for each vaccinated cohort, we calculated relative risk (RR) for each of three models as the ratio of p_1_ (the risk of having developed TB [a notification] or the risk of having developed culture-confirmed TB or the risk of having developed TB meningitis) to p_2_ (the risk observed in the non-vaccinated cohort). The confidence interval (CI) for RR was calculated taking the natural logarithm of the estimate of RR as
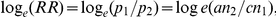
where “a” was the estimated number of TB cases in a given vaccinated cohort, “n_1_” was the number of births in the vaccinated cohort, “c” was the number of cases in the non-vaccinated cohort, and “n_2_” was the number of births in the non-vaccinated cohort. The standard error (SE) of the natural logarithm of RR was estimated as
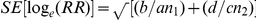
where “b” was the number of non-cases in the vaccinated cohort and “d” was the number of non-cases in the non-vaccinated cohort [12].

The 95% CI for log_e_ (RR) was computed as log_e_ (RR) = ±1.96 SE, and the 95% CI for RR was obtained by taking anti-logarithms. Finally, BCG prevention effectiveness was calculated as (1−RR)×100. Prevention effectiveness was calculated for each of the three models: clinical TB, laboratory-confirmed TB, and TB meningitis. The lower boundary for the 95% CI for prevention effectiveness was calculated as the upper limit of the 95% CI of the RR, and the upper boundary for the 95% CI for prevention effectiveness was calculated as the lower limit of the 95% CI of RR. All p-values were calculated as two-tailed.

Kazakhstan has 16 administrative territories: two cities and 14 oblasts (provinces). To determine if there were regional variations in prevention effectiveness, high TB prevalence oblasts (≥4.02/1,000; range: 4.03–4.34) were compared collectively with low TB prevalence oblasts (≤2.99/1,000; range: 1.69–2.95) with respect to RR and prevention effectiveness of the three BCG vaccines for the entire 3-year follow-up period in the four different cohorts. High prevalence oblasts included Atyrauskaya, Kyzylordinskaya, Mangistauskaya, West Kazakhstan, and Zhambylskaya oblasts; low prevalence oblasts included Almatinskaya, East Kazakhstan, North Kazakhstan, and South Kazakhstan oblasts.

Because of concerns that variations in TB incidence observed between the cohorts may reflect surveillance or TB incidence phenomena across the general population, TB notification data for older children and adults were examined for each of the years 2002–2007.

## Results

The number of newborns in each cohort ranged from 138,059 to 168,664 in the four respective 7-month periods examined (Table 1).

Cohort A (the non-vaccinated group) served as the comparison group for cohorts B, C, and D (Russian, Serbian, and Japanese vaccines, respectively). Prevention effectiveness based on clinical TB case notifications was 69% for the Japanese BCG, 43% for Serbian BCG, and 22% for Russian BCG (Table 2).

**Table 2:** BCG vaccine prevention effectiveness for clinically defined, radiologically confirmed TB cases, Kazakhstan, 2002–2008.

Cohort (BCG product)	BCG Vaccinated			Non-vaccinated (Cohort A)			RR†	95% CI† for RR		PE† (%)	p-value
	# births	# cases	Risk per 1000*	# births	# cases	Risk per 1000*					
B Russian	138,059	207	1.50	160,970	310	1.93	0.78	0.65	0.93	22	0.005
C Serbian	150,938	165	1.09	160,970	310	1.93	0.57	0.47	0.69	43	<0.001
D Japanese	168,664	102	0.60	160,970	310	1.93	0.31	0.25	0.39	69	<0.001

* Risk calculated for the entire follow-up period (3 years).

† RR, relative risk; CI, confidence interval; PE, prevention effectiveness.

Of the 784 patients in the four cohorts who had specimens cultured for TB, 20 (2.5%) had positive cultures (Table 3). Estimates of prevention effectiveness levels based on laboratory-confirmed TB cases were 92% for the Japanese BCG, 82% for Serbian BCG, and 51% for Russian BCG.

**Table 3:** BCG vaccine prevention effectiveness for culture positive TB cases, Kazakhstan, 2002–2008.

Cohort (BCG product)	BCG Vaccinated			Non-vaccinated (Cohort A)			RR†	95% CI† for RR		PE† (%)	p-value
	# births	# cases	Risk per 1000*	# births	# cases	Risk per 1000*					
B Russian	138,059	5	0.04	160,970	12	0.07	0.49	0.17	1.38	51	0.166
C Serbian	150,938	2	0.01	160,970	12	0.07	0.18	0.04	0.79	82	0.011
D Japanese	168,664	1	0.01	160,970	12	0.07	0.08	0.01	0.61	92	0.002

* Risk calculated for the entire follow-up period (3 years).

† RR, relative risk; CI, confidence interval; PE, prevention effectiveness.

BCG prevention effectiveness based on numbers of TB meningitis case notifications for each vaccinated cohort ranged from 71% to 89% (Table 4). Each vaccine had statistically significant prevention effectiveness for TB meningitis when compared with the outcomes of the non-vaccinated cohort.

**Table 4:** BCG vaccine prevention effectiveness for TB meningitis cases, Kazakhstan, 2002–2008.

Cohort (BCG product)	BCG Vaccinated			Non-vaccinated (Cohort A)			RR†	95% CI† for RR		PE† (%)	p-value
	# births	# cases	Risk per 1000*	# births	# cases	Risk per 1000*					
B Russian	138,059	2	0.01	160,970	10	0.06	0.23	0.05	1.05	77	0.040
C Serbian	150,938	1	0.01	160,970	10	0.06	0.11	0.01	0.86	89	0.009
D Japanese	168,664	3	0.02	160,970	10	0.06	0.29	0.08	1.05	71	0.043

* Risk calculated for the follow-up period specifically used for meningitis cases (21 months).

† RR, relative risk; CI, confidence interval; PE, prevention effectiveness.

**Figure 2:**
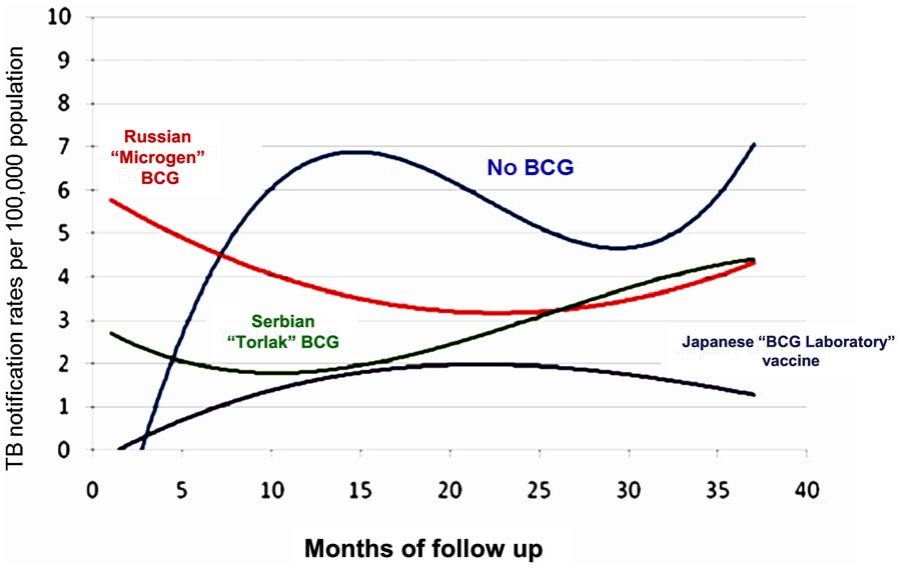
Moving average (2-month) TB notification rate of different birth cohorts born in September–March, by type of BCG administered, Kazakhstan, 2002–2008.

The moving average (2-month) analysis of incidence rate based on clinical case notifications in the cohorts demonstrated a rapid increase in TB incidence during first year of life among non-vaccinated children (Figure 2). The vaccinated cohorts had no comparable increase. Relative to each vaccinated cohort, there was an increased TB risk among the non-vaccinated cohort. The three vaccines differed significantly in prevention effectiveness.

**Figure 3:**
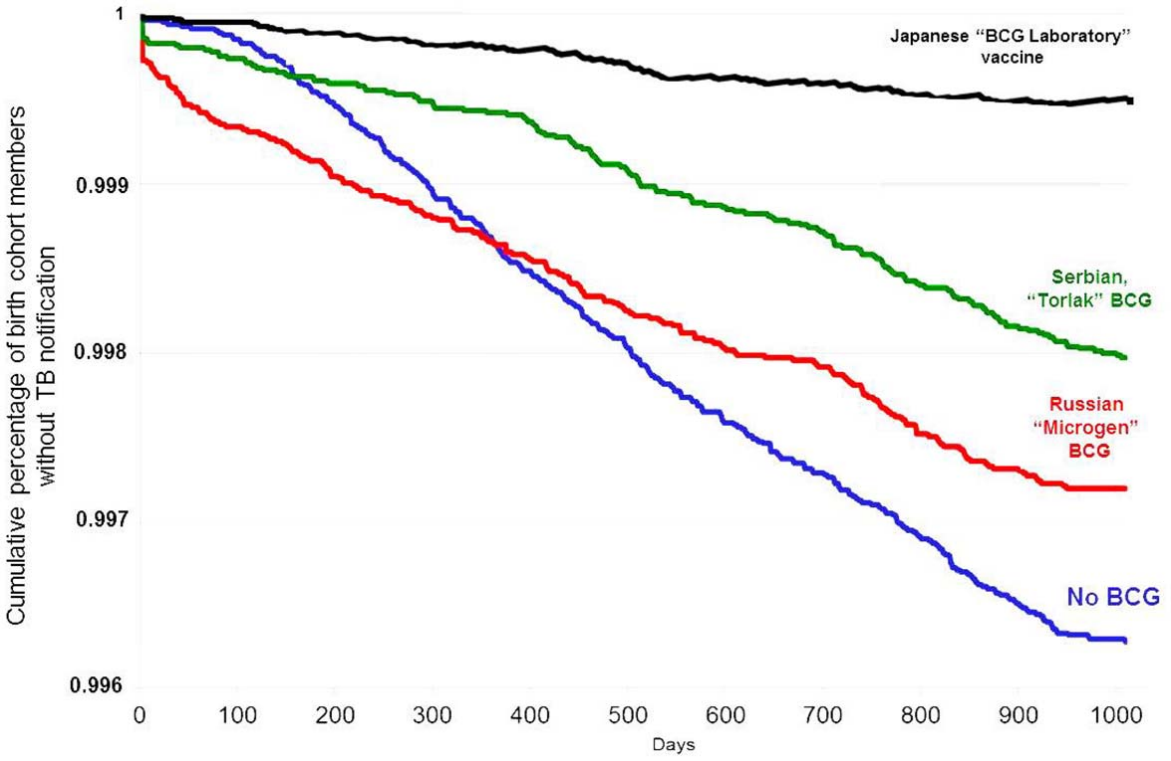
Survivor curve person-year analyses for reported incidence of active TB among different BCG vaccinated and non-vaccinated birth cohorts, Kazakhstan, 2002–2008.

Survivor curve person-year (life table) analyses for TB prevention among the BCG vaccinated and non-vaccinated cohorts demonstrated significant prevention effectiveness for all three vaccines with respect to developing disease resulting in TB case notification (Figure 3). The cumulative percentage of cohort members without TB during the first 1,000 days of follow-up among non-vaccinated children was 0.9964%, but among the cohort vaccinated with the Japanese vaccine, the percentage of cohort members without TB during the first 1,000 days of the follow-up period was 0.9996%, i.e., the vaccinated cohort had 32 more disease-free person-years per 10,000 person-years than the non-vaccinated cohort had.

The prevention effectiveness hierarchy of manufacturers with respect to clinical TB remained the same for the three vaccines when high TB prevalence oblasts were compared collectively to low TB prevalence oblasts (Table 5).

**Table 5:** Relative risk and TB prevention effectiveness of BCG vaccines in different birth cohorts in areas of low (≤2.99/1,000) and high (≥4.02/1,000) reported TB incidence, Kazakhstan, 2002–2008.

Reported TB incidence§	Cohort (BCG product)	BCG Vaccinated			Non-vaccinated (Cohort A)			RR†	95% CI for RR†		PE† (%)
		# births	# cases	Risk per 1000*	# births	# cases	Risk per 1000*				
Low (≤2.99/1000)	B Russian	68,209	61	0.89	80,489	92	1.14	0.78	0.57	1.08	22
	C Serbian	75,333	48	0.64	80,489	92	1.14	0.56	0.39	0.79	44
	D Japanese	84,245	34	0.40	80,489	92	1.14	0.35	0.24	0.52	65
High (≥4.02/1000)	B Russian	26,670	74	2.77	31,711	112	3.53	0.79	0.59	1.05	21
	C Serbian	29,472	65	2.21	31,711	112	3.53	0.62	0.46	0.85	38
	D Japanese	33,397	35	1.05	31,711	112	3.53	0.30	0.20	0.43	70

* Risk calculated for the entire follow-up period (3 years).

† RR, relative risk; CI, confidence interval; PE, prevention effectiveness.

§ High reported TB incidence - five oblasts: Atyrauskaya, Kyzylordinskaya, Mangistauskaya, West Kazakhstan, and Zhambylskaya. Low reported TB incidence - four oblasts: Almatinskaya, East Kazakhstan, North Kazakhstan, and South Kazakhstan.

TB notification data for older children and adults showed little variation across the years examined. For persons aged 15–44 years, the numbers of cases reported for the first time for the years 2002–2007 were respectively 27,054; 30,230; 30,003; 30,202; 28,452; and 27,024. For persons aged 45+ years, the numbers of cases reported for the first time for the years 2002–2007 were respectively 9,194; 10,646; 10,841; 11,451; 10,972; and 10,475.

## Discussion

The study data demonstrate positive but differing prevention effectiveness for clinical TB (22%, 43%, 69%) in Kazakhstan for BCG vaccines from three producers, comparing outcomes of three vaccinated cohorts of infants with those of a non-vaccinated cohort; during the time-frames studied, modification of BCG sources and a 7-month suspension of BCG administration were the only externally imposed variations in an otherwise unmodified national TB prevention program. The prevention effectiveness hierarchy of manufacturers for clinical TB was the same whether one were evaluating areas of high- or low-TB prevalence. All three BCG vaccines were also protective but with differing effectiveness observed for culture-confirmed TB and for TB meningitis.

Published case-control studies have yielded widely disparate measures of protection from different BCG vaccines, ranging from no reduction [13] to an 83% reduction [14] in TB incidence. In 1994, George Comstock proposed using a controlled methodology to measure relative vaccine efficacy changes in geographic areas in which the BCG vaccine had been recently changed [15], noting that “…countries that have a need for vaccination and that vaccinate at birth could be recruited to use their own vaccine in even years and a different vaccine in odd years without making any other changes in their vaccination programs. Merely accumulating reported cases and deaths among persons born in these even and odd years would reflect effects of the vaccines.” The use of three different BCG vaccines within a relatively short period of time and the interruption of a BCG vaccination program yielded non-vaccinated and vaccinated cohorts that provided a unique opportunity to evaluate the effectiveness of BCG vaccines in a model that resembled the one proposed by Comstock: the experience of three different infant cohorts, each vaccinated with a different BCG product, could be compared with that of a non-vaccinated cohort; very large numbers included in each cohort (range: 138,059–168,664) provided adequate sample sizes for statistical analysis; the retrospective approach to identifying study cohorts avoided the ethical quandary of using a vaccine strategy in infants that did not include BCG; the vaccinated populations studied were newborns and relatively mycobacteria-naïve, in comparison to older children or adults who may have acquired mycobacterial immunity and whose inclusion might have reduced the observed BCG effectiveness [16]; and the control (non-vaccinated) cohort was drawn from the same population from which the comparison (vaccinated) cohorts came.

Potential limitations of this study need to be considered. First, the methodology used was a retrospective cohort study, but the best method for determining the protective effect of a vaccine would be a prospective, randomized, double-blind, placebo-controlled trial; such studies are rarely performed because of their difficulty and expense, and the intent of this study was to make a relatively inexpensive effort to analyze previously gathered data to shed light on the prevention effectiveness of each vaccine preparation. In addition, a prospective trial that employed a vaccine strategy arm that did not include BCG could potentially be considered unethical, because BCG confers protection against meningeal and disseminated TB disease in childhood [1], [17], [18]. Second, to interpret the measured effectiveness of BCG vaccine in a program as a reflection of vaccine efficacy, one would have to assume that each cohort of vaccinated and non-vaccinated children was similar with respect to potential risk factors for TB exposure and infection. Although our comparison cohorts were consistent in time and place, risk factors that could have varied and affected the measures of prevention effectiveness observed between cohorts included accuracy of diagnosis, exposure to environmental mycobacteria, vaccine virulence, and transmissibility of locally endemic TB strains, household exposure risk, and population genetics. Indeed, studies using the same BCG strain in different countries have yielded varying levels of protection [19]. Although there was no reason to assume that exposure to circulating non-tuberculosis mycobacterium strains, transmissibility of locally endemic TB strains, diagnostic methods, or population genetics and immunity would have appreciably varied between the cohorts chosen, there were small but progressive year-to-year decreases in population burden of TB disease that could have had concomitant minor effects on the potential risk of household exposure (and hence risk of primary infection, as opposed to reactivated disease) for those cohorts. Third, most cases were clinically diagnosed; this is commonly the case in children, but pediatric clinical diagnoses are more subjective and subject to more variation. Data for culture positive cases were analyzed separately, but the numbers of culture-positive cases were sparse and the CIs for prevention effectiveness for culture-positive disease were wide. Similarly, when data for TB meningitis cases were analyzed, the numbers of TB meningitis cases were also sparse and the CIs for prevention effectiveness for TB meningitis were wide. Fourth, small variations were noted in TB notification data that could have reflected differences in relative risk of exposure of the cohorts. Fifth, in a differential diagnosis in the absence of laboratory confirmation, clinicians may be influenced away from a TB diagnosis because of their expectations of BCG effectiveness and their knowledge of a child's vaccination status, which would contribute to overestimates of prevention effectiveness. However, clinicians were unlikely to know which BCG a child received; thus, expectations of BCG effectiveness would not have had an effect on the differences in prevention effectiveness observed between the vaccines. Sixth, because the non-vaccinated cohort was the comparison group for each of the vaccinated cohorts, lack of availability of data regarding any catch-up vaccination that occurred in the non-vaccinated cohort would systematically bias the data that were used in this analysis toward minimizing the true prevention effectiveness of each of the vaccines in equal measure.

Historically, demand for BCG led the Institute Pasteur to distribute the original strain to the world before protocol standards for culture were established [20]. Reduced efficacy has subsequently been reported for strains that have had greater numbers of serial passages, and genetic differences have been noted between strains that may explain the differences in reported prevention effectiveness [21], [22]. Indeed, in a recent randomized trial of three different commonly used BCG vaccine strains, significant differences were noted in the immune responses (numbers of mycobacterium-specific polyfunctional and cytotoxic T cells, and concentrations of Th1 cytokines) induced by the different vaccine strains in newborns [23]; this work provides a potential immunologic basis for the differences in prevention effectiveness of the BCG vaccines observed in our morbidity data.

Evaluation of the effectiveness of BCG programs is important, especially in developing countries, and identification of strains that offer superior protection would have worldwide applicability, especially for developing and transitional economies. Other approaches outlined for improving existing BCG vaccination strategies include modifying strains to express *Mycobacterium tuberculosis* antigens with greater immunogenicity, and using prime-boost strategies (supplemental to BCG administration) with supplemental inoculation with viral vectors encoding *M. tuberculosis* antigens or protein subunits [24], [25]. In countries in which TB incidence is very high, small increments BCG prevention effectiveness could potentially prevent large numbers of TB cases and their attendant high death rate, especially in children.

### Conclusions

Three different BCG vaccine preparations were evaluated using data gathered retrospectively and were found to be effective in preventing TB disease. TB prevention effectiveness of BCGs varies by manufacturer. The prevention effectiveness hierarchy of manufacturers for clinical TB was the same whether one were evaluating areas of high- or low-TB prevalence. Ascertaining the relative effectiveness/efficacy of BCG vaccines from different producers may have implications when setting national and global policy, as use of strains that offer superior protection may be more cost-effective.
